# Assessment of Pictographs Developed Through a Participatory Design Process Using an Online Survey Tool

**DOI:** 10.2196/jmir.1129

**Published:** 2009-02-24

**Authors:** Hyeoneui Kim, Carlos Nakamura, Qing Zeng-Treitler

**Affiliations:** ^2^Decision Systems GroupBrigham and Women’s HospitalHarvard Medical SchoolBostonMAUSA; ^1^Clinical Informatics Research and DevelopmentPartners Healthcare SystemBostonMAUSA

**Keywords:** Discharge instructions, patient communication, pictograph, readability, health literacy

## Abstract

**Background:**

Inpatient discharge instructions are a mandatory requirement of the Centers for Medicare and Medicaid Services and Joint Commission on Accreditation of Healthcare Organizations. The instructions include all the information relevant to post-discharge patient care. Prior studies show that patients often cannot fully understand or remember all the instructions. To address this issue, we have previously conducted a pilot study in which pictographs were created through a participatory design process to facilitate the comprehension and recall of discharge instructions.

**Objective:**

The main objective of this study was to verify the individual effectiveness of pictographs created through a participatory design process.

**Methods:**

In this study, we included 20 pictographs developed by our group and 20 pictographs developed by the Robert Wood Johnson Foundation as a reference baseline for pictographic recognition. To assess whether the participants could recognize the meaning of the pictographs, we designed an asymmetrical pictograph–text label-linking test. Data collection lasted for 7 days after the email invitation. A total of 44 people accessed the survey site. We excluded 7 participants who completed less than 50% of the survey. A total of 719 answers from 37 participants were analyzed.

**Results:**

The analysis showed that the participants recognized the pictographs developed in-house significantly better than those included in the study as a baseline (*P*< .001). This trend was true regardless of the participant’s gender, age, and education level. The results also revealed that there is a large variance in the quality of the pictographs developed using the same design process—the recognition rate ranged from below 50% to above 90%.

**Conclusions:**

This study confirmed that the majority of the pictographs developed in a participatory design process involving a small number of nurses and consumers were recognizable by a larger number of consumers. The variance in recognition rates suggests that pictographs should be assessed individually before being evaluated within the context of an application.

## Introduction

	Centers for Medicare and Medicaid Services (CMS) and the Joint Commission on Accreditation of Healthcare Organizations (JCAHO) require that, upon discharge, all inpatients receive detailed instructions for self-care management [1,2]. The delivery of discharge instructions to a patient is the culmination of a complex process. The planning and assessment of discharge instructions begin with the patient&rsquo;s first visit and continue until the patient leaves the hospital. Typically, patients receive the instructions both orally and in writing. Upon discharge, a nurse reads and explains the written instructions to make sure the patient understands them. The patient keeps a copy of the written instructions for later consultation. The instructions include all the information relevant to post-discharge patient care.

Despite this duplication of information, some studies show that patients often cannot fully understand or remember all the instructions [3-5]. Research also shows that this lack of comprehension affects patient satisfaction and compliance [6-8]. Comprehension of medical instructions can be especially challenging for people with lower reading skills. The average reading skill level of the adult population in the United States is estimated to be between the 8th and 9th grade level [9]. However, recent studies show that more than half of medical instructions are written at levels higher than the 10th grade [10-12]. A study on Medicare enrollees reported that about 23% of English-speaking enrollees could not adequately understand medical instructions written in English [13].

In addition to the complexity of the instructions, factors such as physical and emotional distress, lack of motivation, and environmental distractions make understanding and remembering instructions challenging to patients. Since these other factors cannot be eliminated at the time of discharge, researchers and clinicians explore different strategies to make the instructions easier to understand. There have been a few studies showing that the addition of pictures can enhance the comprehension of written medical instructions [19]. In a study by Austin et al, comprehension of discharge instructions increased by 1.5 times when pictures were added to the instructions [14]. Mansoor and Dowse [15] reported that comprehension of the correct method for taking a medicine increased from 47% to 93% when pictures were added. The authors also reported that comprehension of the proper times to take a medicine increased from 3% to 73% when pictures were added. The effects of pictures on recall are less conclusive. There have been studies that show no effect [16] as well as studies that show an increase in recall when pictures are added to written instructions [17,18].

While studies have demonstrated that pictographs can improve comprehension, several factors inhibit the use of pictographs in discharge instructions. There exists no standard pictographic language for patient communication, and there has been limited research on how to systematically develop and evaluate pictographs for patient communication. In an attempt to foster the use of pictographs, we are conducting a three-stage project on systematic ways to develop pictographs that are effective for patient communication. In the first stage, we experimented with a participatory design process. In the second and current stage, we assessed the recognition of individual pictographs that were developed by us using a well-established pictographic system as a reference point. In the third stage, we will analyze which syntactic and semantic factors are the best predictors of pictographic recognition. The analysis also involves the identification of the best representation techniques for each category of concept.

### Prior Work

In one of our previous studies, we used a participatory design process to design pictographs to improve comprehension and recall of discharge instructions [20]. We recruited four participants—two nurses and two consumers—to identify discharge instructions that could be improved through pictorial aids and to help design the pictographs. The participants were presented with 38 specific instructions selected from a convenience sample of 30 discharge documents. For each instruction, the participant was asked to consider the following: (1) Is the instruction easy to understand? (2) Will the use of a pictograph make the instruction easier? (3) If a pictograph is recommended, what is the best way to design it?

The nurses suggested that 32 of the 38 instructions would benefit from a pictograph and provided specific instructions for their design. The consumers did not identify any specific instruction, but they believed that, in general, including a pictograph would be helpful.

Due to time and resource constraints, we selected 20 of the 32 instructions as candidates for pictograph design. The initial designs were developed by one investigator in our group and given to the nurses and consumers for review and feedback. Participants were asked to consider whether the meaning of the pictograph was clear, whether the label matched the semantics, and how (if) the pictograph could be improved.

To evaluate the pictographs, we composed two mock-up discharge instruction documents, A and B, based on two different medical scenarios. For each mock-up we created two versions: a text-only version reflecting a typical discharge instruction sheet and a pictograph-enhanced version using the images we developed. The mock-up documents were tested on a convenience sample of 13 subjects. Each subject was randomly assigned to one of two groups. Group I received the text version of A and the pictograph-enhanced version of B. Group II received the text version of B and the pictograph-enhanced version of A.

In evaluation, each participant was presented with an instruction and was asked to assume the role of a patient being discharged while the investigator played the role of a discharge nurse. Immediate recall was measured by asking the participant to write down what he or she remembered immediately after reading and reviewing the instruction. Delayed recall was collected, also in writing, after 5 days. The same procedure was used for the second instruction. The correctness of participants’ answers was not conditional to verbatim recall. If the original instruction was “avoid swimming” and a participant wrote “do not swim,” that item would be scored as correct. Each item in the participants’ responses was scored as correct, partially correct, or wrong. For example, if a participant wrote down “take this drug two hours before eating dairy food” and the original instruction was “take this drug two hours before or two hours after eating dairy food,” that item would receive a partial score. The interviewing nurse was responsible for rating the correctness and completeness of each recalled instruction.

Both immediate and delayed recall rates were higher for the pictograph-enhanced instruction. The mean immediate recall rates were 44.28% (sd16.14%) for the text-only version and 53.51% (sd17.53%) for the pictograph-enhanced condition. The mean delayed recall rates (5 days) were 27.31% (sd14.09%) for the text-only condition and 33.03% (sd15.95%) for the pictograph-enhanced condition. Mixed factor linear regression analysis found statistically significant effects (*P*< .001) of presentation format (text versus text and pictograph) on the recall rate. The effect of case (A or B) on the recall rate was not significant (*P*= .49). The results suggested that the pictographs we developed were effective in improving the recall of discharge instructions. These results are consistent with existing research. Kitching found that outpatients usually forget about 50% of their doctor’s instructions within 5 minutes of leaving the doctor’s office [21]. In another study, by Logan et al, only 37% of patients could correctly recall their diagnosis, treatment, and follow-up plans immediately after being discharged from the emergency room [22].

### Objectives

Although the pictographs in the study described above were developed through a participatory design process and were shown to improve instruction recall, we considered it important to conduct further studies to assess the individual pictographs. Pictographs used in health care are often empirically designed and seldom quantitatively evaluated [23]. Without a systematic design approach, pictographs are less likely to be useful or helpful. For example, Hwang et al reported that the addition of a certain set of icons did not improve patients’ comprehension of medication labels [5]. Rigorous and quantitative evaluations are needed because user preference alone is not a reliable measure of the effectiveness of pictographic communication [23].

The main objective of this study was to verify the recognition of individual pictographs created through a participatory design process. We hypothesized that comprehension and recognition would be affected by the individual characteristics of the pictographs as well as by the characteristics of the intended audience (demographic factors).

## Methods

### Materials

In this study, we included 20 pictographs developed by our research group in a prior study and 20 pictographs developed by the Hablamos Juntos project, funded by the Robert Wood Johnson Foundation (RWJF) [24]. It is important to emphasize that we are not using the RWJF pictographs for direct comparison but rather as a reference point for pictographic recognition. The two pictographic systems are indeed very different in nature. The RWJF pictographs were designed to help visitors navigate health facilities, whereas our pictographs were designed to help patients understand discharge instructions. Wayfinding pictographs, like traffic signs, are supposed to be graphically as simple as possible. Pictographs depicting medical instructions are necessarily more elaborate as the messages they are supposed to convey are far more complex. We chose the RWJF pictographs as a comparison reference because they are one of the most successful examples in the health care domain, where systematic initiatives in pictographic communication are few. See the Multimedia Appendix for the complete list of pictographs.

Each participant was asked to identify 10 pictographs developed by our group and 10 pictographs developed by RWJF. The pictographs developed in-house were color images, whereas those collected from RWJF were black-and-white.

To assess whether the participants could recognize the meaning of the pictographs, we designed an asymmetrical pictograph–text label-linking test. For each pictograph, we provided 20 labels. The correct label for the pictograph was presented among the 20 labels 70% of the time. The order of the pictographs and the text labels was randomized. The participants were asked to select either a matching label or “none” if no label appeared to match. Thus, if a participant made random selections, there would be less than a 5% chance of getting the right answer. On the other hand, since recognition is an easier cognitive task than recall, this is not an overly challenging task. When the participant picked the “none” option, he or she was given the opportunity to suggest an accurate label, although suggesting a new label was not required.

**Figure 1:**
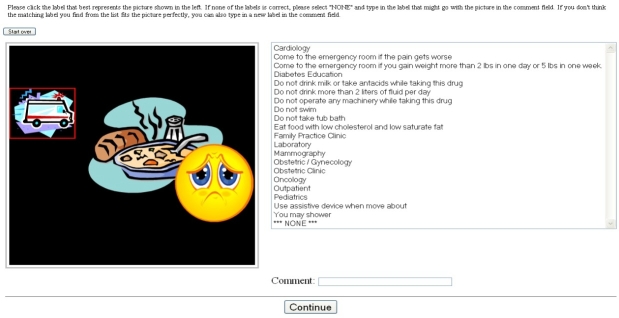
Screenshot of the Web-based survey tool for pictograph evaluation

To facilitate the anonymous survey study, we created a Web-based tool. A screenshot of the survey tool is shown in Figure 1. No adaptive questioning procedures were used. We did not perform consistency or completeness checks before the questionnaire was distributed.

#### Sampling

We worked with a convenience sample. After obtaining approval from the Institutional Review Board, the survey was advertised via email to colleagues and acquaintances of the authors. Participation was voluntary and anonymous. No monetary or non-monetary incentives were offered. To evaluate the effects of demographic factors on the perception of pictographs, the survey also asked six demographic questions about age bracket, race, ethnicity, education level, and the continent where the participant grew up.

#### Analysis

The participants’ answers were scored according to whether they were correct or not. The scores were also weighted to reflect the difficulty of providing a correct answer, depending on the presence or absence of a correct label (Table 1). “Correct label selected” involves scanning the list; it is a self-terminating search (weight: 1). “None selected” requires the subject to serially review the list; it is an exhaustive search—all items need to be considered (weight: 2). “Correct term added” requires the spontaneous generation of an appropriate label (weight: 3).

**Table 1:** Scoring scheme for the label-linking test (weights are in parentheses)

	Actual Label Present	Actual Label Absent
Correct label selected	1 (1)	N/A
Incorrect label selected	0 (1)	0 (1)
None selected, correct term not added	0 (1)	1 (2)
None selected, correct term added	N/A	1 (3)

We calculated weighted average scores for every pictograph, for the different demographic groups, and for the two pictograph sources (those developed in-house versus those developed by RWJF). In addition, we used the Wilcoxon two-sample test to examine the effect of demographic factors on recognition of the pictographs. For some demographic variables, several small categories were collapsed into one group, and the cases with missing demographic information were omitted from the analysis.

## Results

Survey data were collected for the 7 days following the email invitation. A total of 44 people accessed the survey site. We excluded 7 participants who completed less than 50% of the survey. A total of 719 answers from 37 participants were used for the analysis. The number of answers obtained per pictograph varied from 11 to 25 due to incomplete survey sessions. The majority of the study participants were highly educated, Caucasian men between 18 and 39 years old who grew up in North America, Asia, or Europe (Table 2).

**Table 2:** Demographic characteristics of the survey participants

Demographic Characteristic	No.	Percent
**Gender**		
Male	22	60
Female	13	35
Unknown	2	5
**Age**		
18-29	14	38
30-39	14	38
40-49	4	11
50-59	3	8
Unknown	2	5
**Race**		
Asian	11	30
Caucasian	21	57
Other	1	2
Unknown	4	11
**Ethnicity**		
Hispanic	3	8
Non-Hispanic	30	81
Unknown	4	11
**Education**		
College, vocational school	1	3
University	3	8
Graduate school	31	84
Unknown	2	5
**ContinentGrew Up In**		
Asia	10	27
Europe	8	22
Middle or South America	2	5
North America	12	32
Unknown	5	14

**Figure 2:**
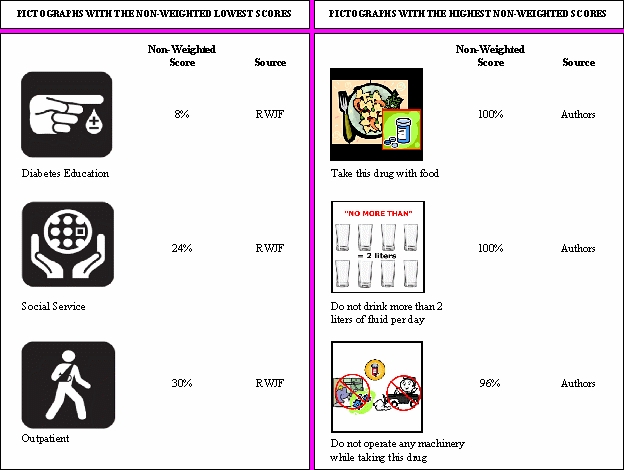
Pictographs with the lowest and highest recognition scores

The majority of the pictographs were recognized by most participants. The average weighted recognition score was 71.81% for the pictographs developed in-house and 57.27% for the RWJF pictographs. However, 7 out of the 40 pictographs obtained scores below 50%; 2 were developed in-house and the other 5 were developed by RWJF. Non-weighted scores measure how successful the participants were in the linking task. Weighted scores take into consideration whether or not participants were able to infer the meaning of the pictographs in cases where the correct label was not present in the list. None of the pictographs received a perfect weighted score. However, 2 of the pictographs developed in-house (“Take this drug with food” and “Do not drink more than 2 liters of fluid per day”) were successfully connected to the right label (or the “none” option) by all participants. The 3 pictographs with the highest and the 3 pictographs with the lowest non-weighted scores are presented in Figure 2.

The weighted average scores were calculated and compared by demographic groups and pictograph source (Table 3). The analysis showed that the participants recognized the pictographs developed by the authors significantly better than those developed by RWJF (*P*< .001). This trend was true regardless of the participant’s gender, age, and education level. However, this trend did not hold for non-Caucasian groups. The weighted average score did not show statistically significant differences between genders, age groups, ethnicities, and education levels, which may be partially attributed to the small number of participants in some demographic groups.

**Table 3:** Weighted recognition scores by demographics and sources

	All	By Source		*P*^a^
		Authors	RWJF	
All (n = 37)	.80	.87	.73	< .001^b^
**Gender (n = 33)**				
Female	.78	.86	.70	< .001^b^
Male	.82	.88	.76	.006^b^
*P* value	.52	.74	.41	
**Age (n = 35)**				
< 40	.81	.87	.74	.001^b^
≥ 40	.80	.89	.71	.006^b^
*P* value	.93	.79	.67	
**Race (n = 33)**				
Non-White	.69	.76	.63	.07
White	.87	.93	.80	< .001^b^
*P* value	.004^b^	.003^b^	.03^b^	
**Ethnicity (n = 33)**				
Hispanic	.92	.97	.86	^c^
Non-Hispanic	.79	.86	.73	< .001^b^
*P* value	^c^	^c^	^c^	
**Education (n = 35)**				
Less than graduate school	.76	.83	.68	^c^
Graduate school	.81	.88	.74	< .001^b^
*P* value	^c^	^c^	^c^	
**Place Grew Up In (n = 32)**				
Other than North America	.75	.82	.68	.002^b^
North America	.88	.93	.83	.03^b^
*P* value	.04^b^	.07	.06	

^a^Tested between the sources.

^b^The mean differences are significant at 95% significance level.

^c^Statistical tests were not conducted due to large differences between group sizes.

### Recognition of Individual Pictographs in Relation to Underlying Concepts and Representation Strategies

**Figure 3:**
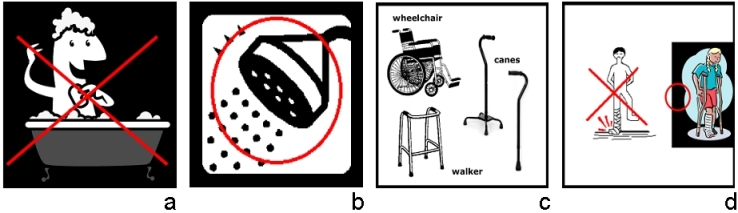
(a) Do not take tub bath; (b) You may shower; (c) Use assistive device when moving about; (d) Do not put your weight on your wounded leg

We made a few observations when analyzing the recognition rate of individual pictographs in relation to their underlying concepts and representation strategies. Most notably, the recognition of pictographs tended to decrease when a person is not included in the picture. For example, 19 out of 22 participants (86%) were able to tell whether the correct label was present or not in the linking test for the “Do not take tub bath” instruction, which included a person in the tub. However, only 6 out of 11 participants (55%) were able to do so with the instruction “You may shower,” which depicted only the shower but no person (see Figure 3). Similarly, 13 out of 17 participants (76%) successfully completed the linking test for the instruction “Do not put your weight on your wounded leg,” whereas 10 out of 15 participants (67%) were successful with the instruction “Use assistive device when moving about,” which did not include any person. These results are consistent with prior research showing that people prefer pictures that include people like themselves [25]. Moreover, the depiction of isolated objects may fail to convey the concept of a verb, which is essential for the comprehension of an instruction.

**Figure 4:**
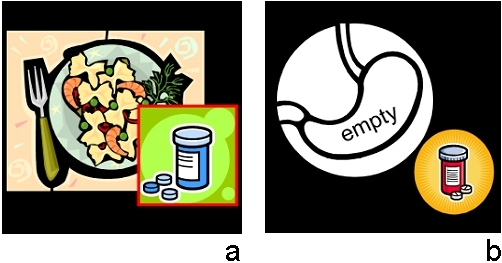
(a) Take this drug with food; (b) Take this drug on an empty stomach

Another observation is that “presence” is easier to represent and understand than “absence.” Take the examples of the pictographs representing “Take this drug with food” and “Take this drug on an empty stomach” (see Figure 4). The former can be easily represented by any depiction of a meal, examples of food, etc. The latter representation is more challenging, so much so that the designer resorted to the use of words to convey the concept of “emptiness.” All 25 participants (100%) successfully completed the linking part of the test for the former, whereas 21 out of 25 participants (83%) did so for the latter. Even with the addition of a verbal descriptor, the concept of “emptiness” was less recognizable than its counterpart.

**Figure 5:**
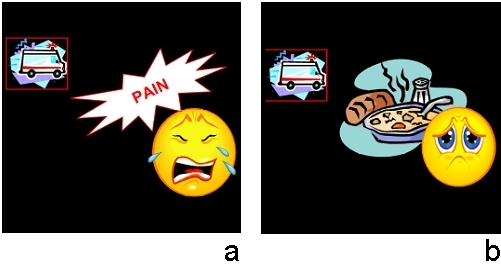
(a) Come to the emergency room if the pain gets worse; (b) Come to the emergency room if you cannot eat food

 A third observation is that abstract concepts are very difficult to represent and understand without the use of some conventions (most notably, words). Take the example of the pictographs “Come to the emergency room if the pain gets worse” and “Come to the emergency room if you cannot eat food” (see Figure 5). In the first example, the pictograph was complemented by the word “pain.” In the second, the reader must infer the idea of an “eating difficulty” without any verbal support. For the former, 9 out of 12 participants (75%) successfully completed the linking part of the test for the meaning of the pictograph, whereas 9 out of 14 participants (63%) did so for the meaning of the latter. It is important to emphasize here that the inclusion of words in pictographs may be less helpful for audiences with poor reading skills. It can be even detrimental if the final audience cannot read English at all. Thus, the inclusion of words in pictographs has to be carefully considered against the assumed reading skills of the intended audience.

## Discussion

### Principal Results

This study confirmed that the majority of the pictographs developed in a participatory design process involving a small number of nurses and consumers were recognizable by a much larger number of consumers. Because RWJF pictographs and our pictographs differ in content and color scheme, a direct comparison between the two is not warranted. However, the RWJF pictographs have been evaluated by over 300 multilingual testers from four language groups [23]. The average recognition score of RWJF pictographs provides a reference point in evaluating the pictographs we developed.

The results also revealed that there is a large variance in the quality of the pictographs developed using the same design process—the recognition rate ranged from below 50% to above 90%. This suggests that pictographs should be assessed individually before being evaluated within the context of an application. Even with the limited number of participants in this study, we observed statistically significant effects of some demographic variables on the recognition of certain pictographs. We thus conclude that assessment tests should always be conducted to ensure that the pictographs used to enhance discharge instructions are appropriate for a diverse patient population.

Both theoretical and empirical studies indicate that the interpretation of pictures is culturally mediated [25-27]. That is, pictures are not universally understood. They carry many cultural components that affect their interpretation. The break down of our results by demographic factors is consistent with those studies. The only differences that were statistically significant were race and continent of origin. Both factors are strongly related to cultural differences. Although education has also been identified as an important factor, we could not analyze it statistically due to large differences between group sizes.

### Limitations

Given our small sample size, we worked with less than optimal statistical power to detect differences in comprehension among the demographic groups. Furthermore, participants in this study were mostly highly literate people. Therefore, we would expect some variation in the ratio of comprehension for populations with much lower literacy levels, which are the people who would benefit the most from the use of pictorial communication.

Because the online survey involved recognition rather than recall, we were not able to capture the potential variations in the interpretation of the pictographs that people may have. Because visual communication is intrinsically less coded than verbal communication, it may often fail to convey certain linguistic intricacies. For example, a pictograph meant to express the warning “Do not take this drug with milk or antacid” could as well be interpreted as “Do not drink milk or antacid while taking this drug.” However, since the pictographs are supposed to complement rather than replace written text, this would not invalidate the relevance of our results.

We have made some observations on the recognition of individual pictographs in relation to their underlying concepts and representation strategies. These observations are still preliminary at this point. More in-depth analyses involving larger samples are indeed necessary. Such analyses are currently being conducted by our research group but are still incipient to be presented here. More information on our follow-up work is offered in the next section.

### Future Directions

In regard to sampling procedures, future studies will achieve more robust outcomes by focusing on more representative and vulnerable populations. Existing research has shown that the vulnerable or at-risk groups are those with less than a high school education, those with racial/ethnic minority status, and those who are over the age of 65 [28].

**Figure 6:**
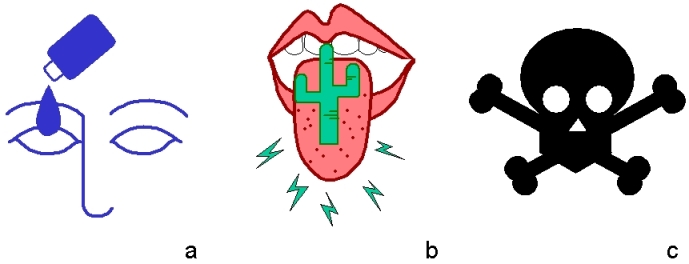
(a) Example of composition by visual similarity: eye drops; (b) Example of composition by semantic association: dry mouth; (c) Example of composition by convention: poison

In regard to the relative recognition levels of the pictographic system we are developing, the results of this study were encouraging. However, they do not quite answer the question of what makes a pictograph easier or harder to understand. To address that concern, we have started the third stage of our project in which we analyze the degree to which pictographs match the concepts they are supposed to depict. This is a taxonomical study in which we adapt discourse analysis techniques to classify pictographs according to their syntactic and semantic design principles. Based on our preliminary analyses, we have found, for instance, that pictographs can relate to concepts by visual similarity, semantic association, or convention (see Figure 6). Pictographs created by similarity tend to be easier to interpret because they actually resemble the concept being depicted. Pictographs developed through semantic association are less reliable because their interpretation depends on the identification of what kind of analogy is being employed (as in the case of a picture of a cactus on a tongue to indicate “dry mouth”). Pictographs that relate to a concept by convention represent a trade-off: those familiar with the convention being used can understand the pictograph instantly, whereas those who are unfamiliar will probably not understand the pictograph at all (as in the case of a skull indicating “poison”). Pictographs created by convention are, thus, the least robust against cultural differences. This is just one example of the many forms of categorization we are employing to produce a taxonomic model that will help us predict the relative recognition of individual pictographs.

## Multimedia Appendix

Pictographs used in the study

## Abbreviations

CMS: Centers for Medicare and Medicaid Services
JCAHO: Joint Commission on Accreditation of Healthcare Organizations
RWJF: Robert Wood Johnson Foundation
