# Supplementary material for: Assessment of Pictographs Developed Through a Participatory Design Process Using an Online Survey Tool
Source: J Med Internet Res. 2009 Feb 24;11(1):e5. doi: 10.2196/jmir.1129 (PMC2762769; doi:10.2196/jmir.1129)
Supplement: Supplementary file 1 [file jmir_v11i1e4_app1.pdf]

Multimedia Appendix

Figures 1 to 20: Pictographs developed in-house

1 Use assistive device when move about

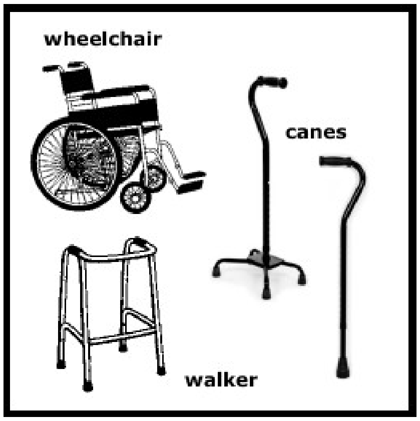

2 Do not put your body weight on your wounded leg

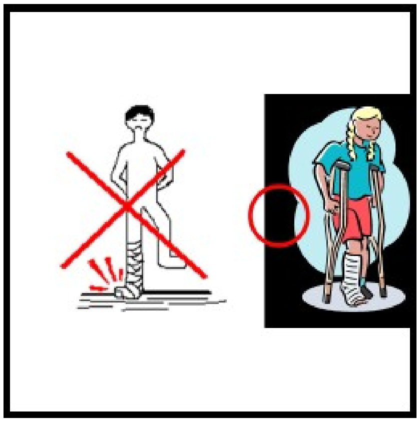

3 Call your doctor if you see drainage from the wound

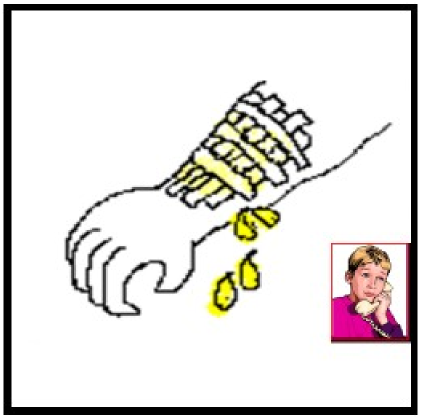

4 Take this drug 1 hour before meal or 2 hours after meal

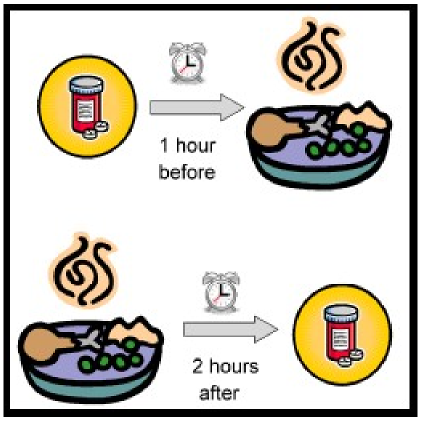

5 Take this drug on an empty stomach

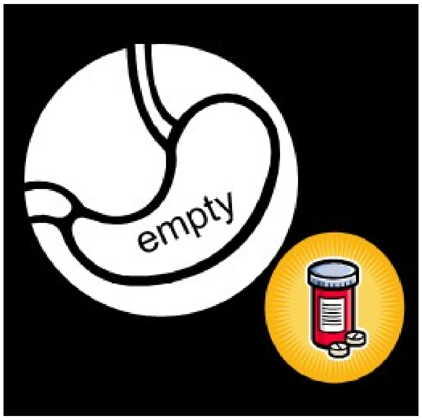

6 Take this drug with food

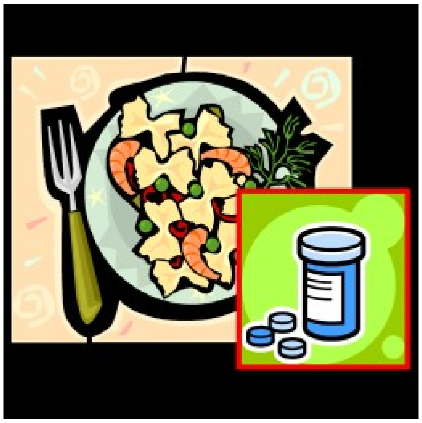

7 Do not eat grapefruit while taking this drug

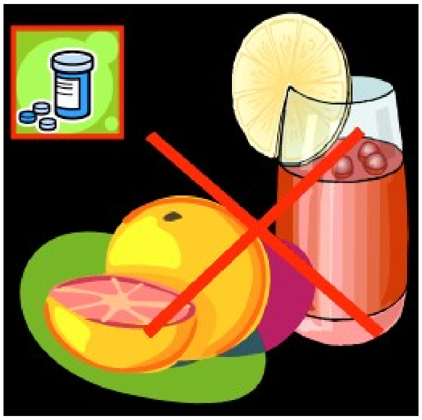

8 Take this drug 2 hours before or 2 hours after eating dairy food

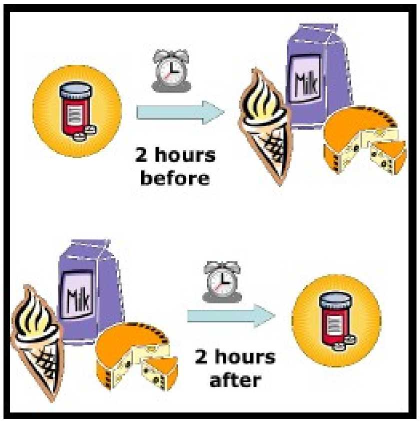

9 Do not drink milk or take antacids while taking this drug

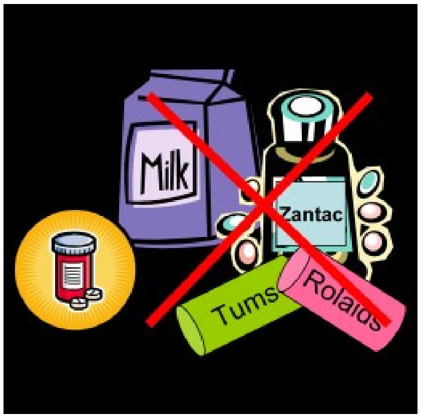

10 Do not operate any machinery while taking this drug

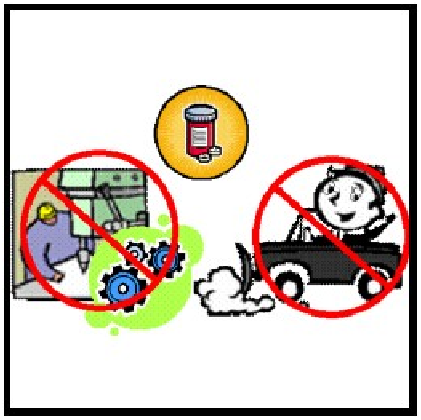

11 Do not take tub bath

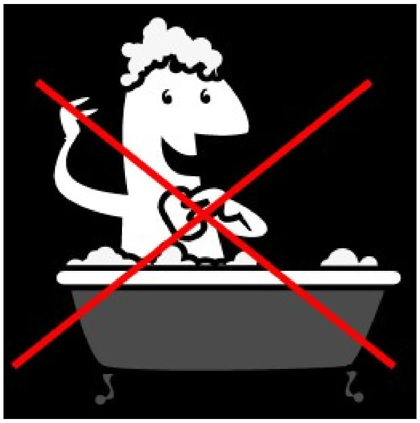

12 Do not swim

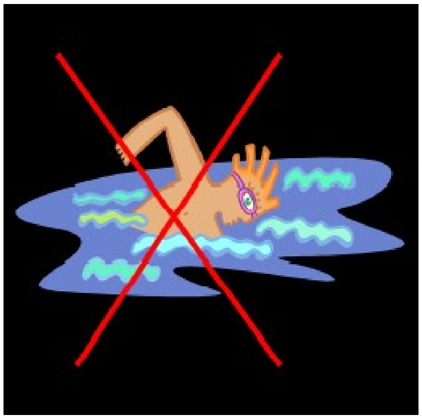

13 Eat food with low cholesterol and low saturate fat

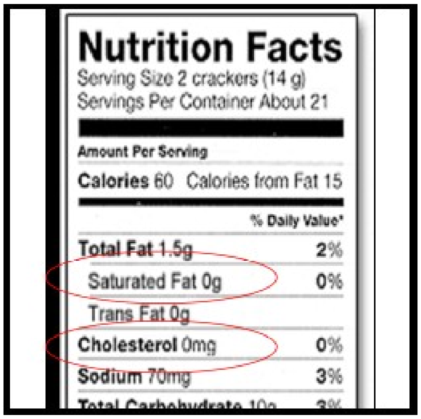

14 Do not drink more than 2 liters of fluid per day

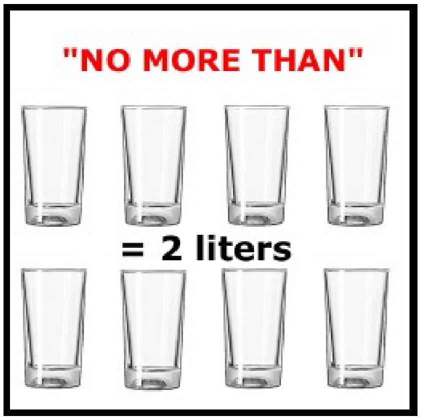

15 You may shower

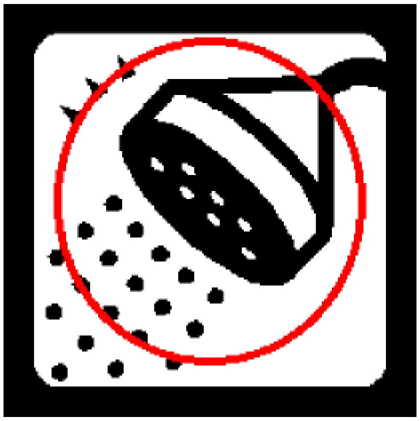

16 Always wear the brace except lying down

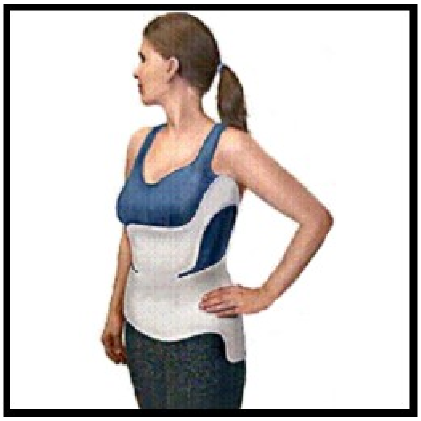

17 Come to the emergency room if you have fever greater than 101.5F

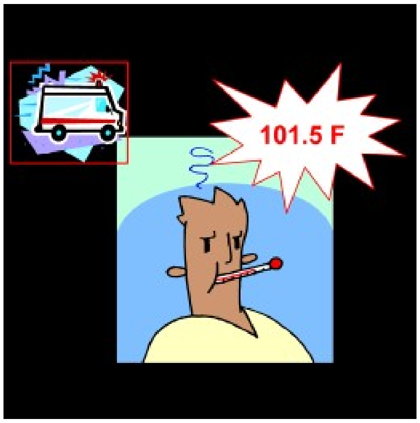

18 Come to the emergency room if you cannot eat food

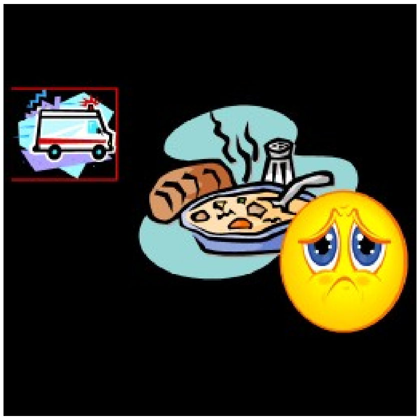

19 Come to the emergency room if the pain gets worse

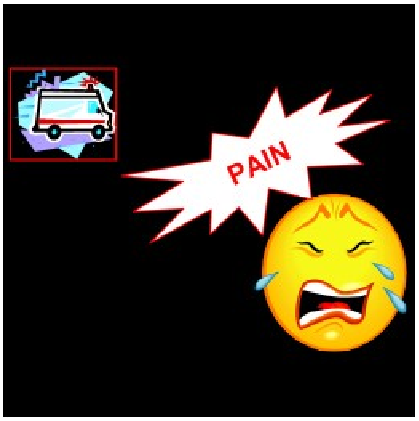

20 Come to the emergency room if you gain weight more than 2 lbs in one day or 5 lbs in one week

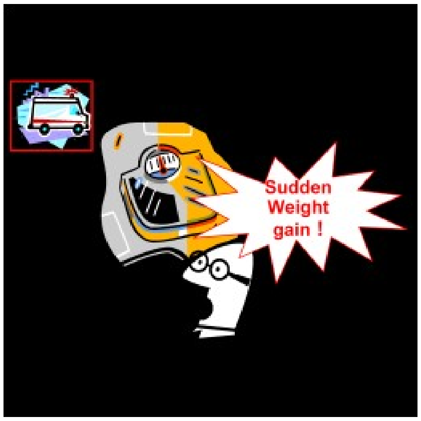

Figures 21 to 40: Universal symbols developed by the *Hablamos Juntos* project, funded by the *Robert Wood Johnson Foundation**

21 Cardiology

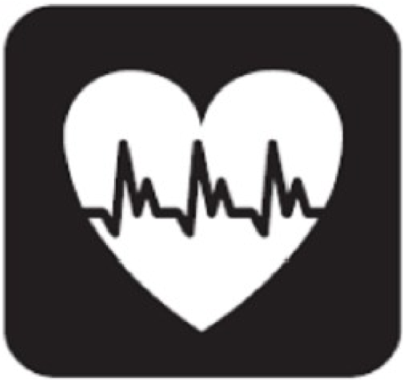

22 Diabetes Education

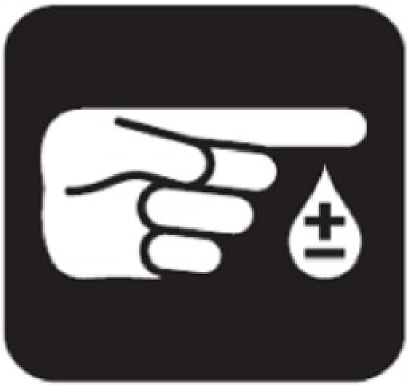

23 Family Practice Clinic

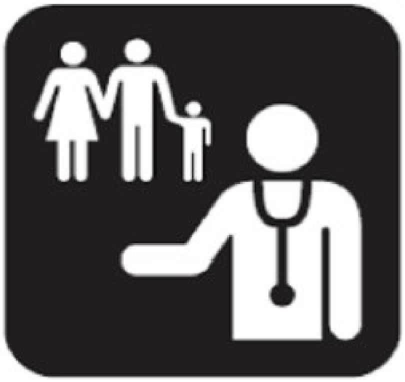

24 Immunizations

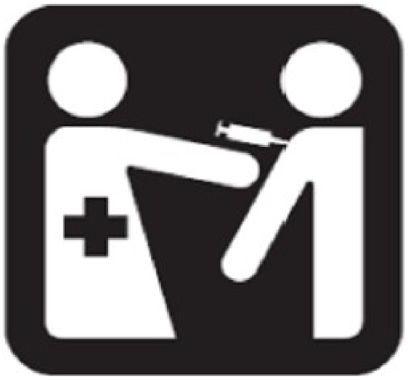

25 Infectious Disease

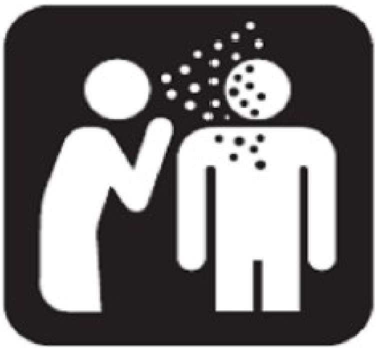

26 Intensive Care

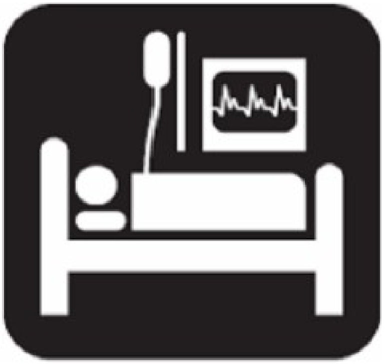

27 Internal Medicine

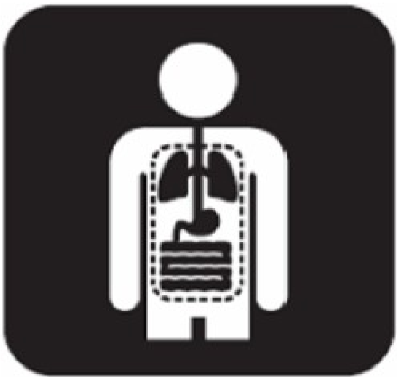

28 Interpretive Services

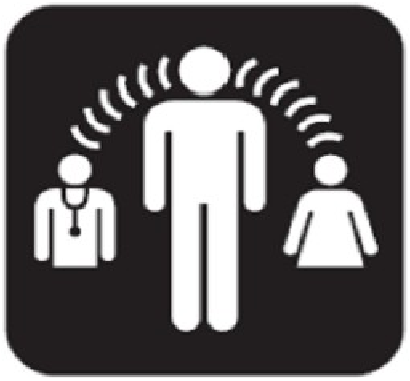

29 Laboratory

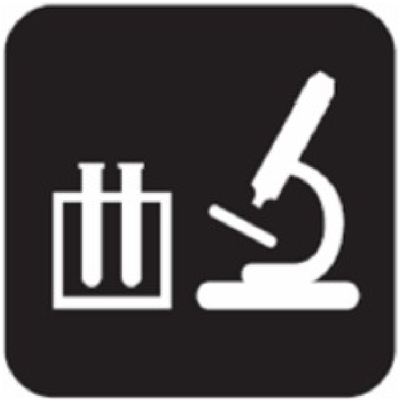

30 Mammography

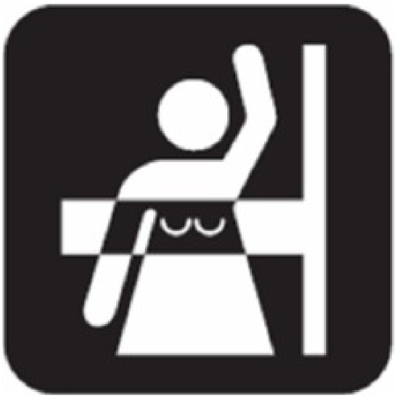

31 Obstetric Clinic

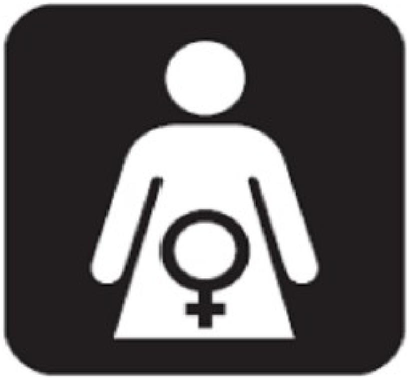

32 Obstetric / Gynecology

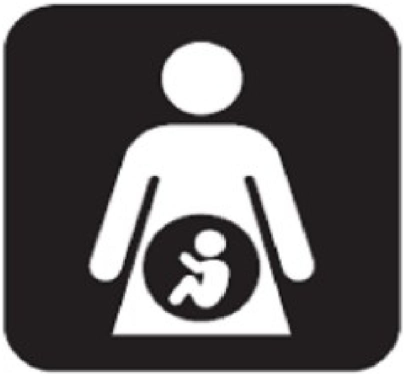

33 Oncology

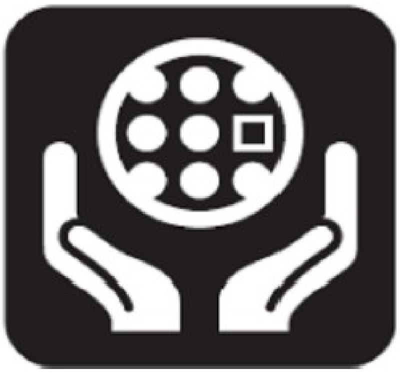

34 Outpatient

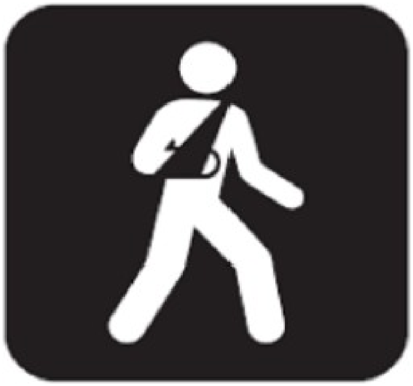

35 Pediatrics

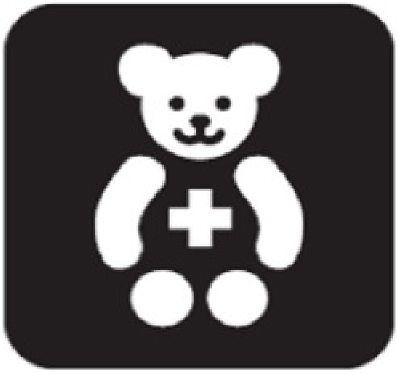

36 Pharmacy

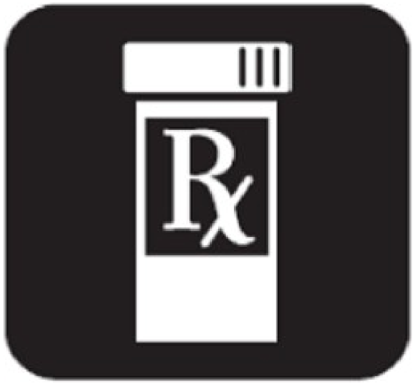

37 Physical Therapy

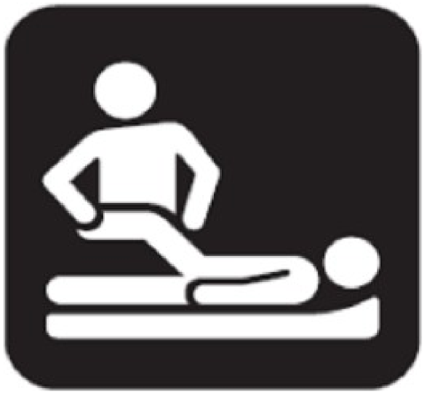

38 Radiology

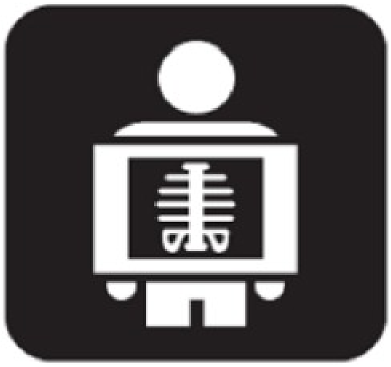

39 Social Service

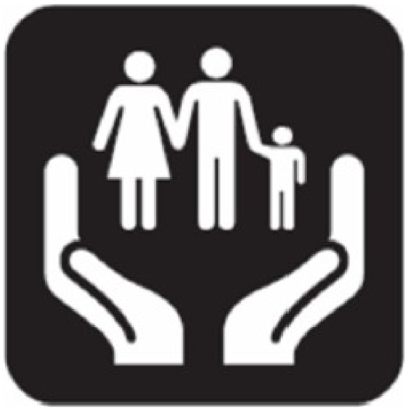

40 Surgery

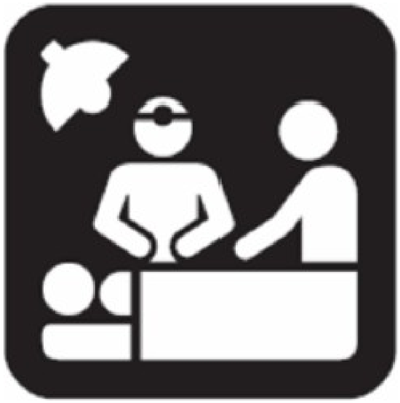

* The following disclaimer is posted on the *Hablamos Juntos* website:
"The symbols and any materials developed to aid in their use are free. Upon completion of the initial set, the symbols were designated as public domain, thanks to the grant of the Robert Wood Johnson Foundation."
Source: http://www.hablamosjuntos.org/signage/symbols/default.using_symbols.asp
